# Exploring the impact of plant genotype and fungicide treatment on endophytic communities in tomato stems

**DOI:** 10.3389/fmicb.2024.1453699

**Published:** 2024-09-25

**Authors:** Luisa Liu-Xu, Liang Ma, Atefeh Farvardin, Pilar García-Agustín, Eugenio Llorens

**Affiliations:** ^1^Biochemistry and Biotechnology Group, Department of Biology, Biochemistry and Natural Sciences, Jaume I University, Castellón de la Plana, Spain; ^2^The Key Laboratory for Quality Improvement of Agricultural Products of Zhejiang Province, College of Advanced Agricultural Sciences, Zhejiang A&F University, Hangzhou, China

**Keywords:** *S. lycopersicum*, tomato, plant genotype, endophytic microbiota, fungi, bacteria, fungicides

## Abstract

This study examines how plant genotype can influence the microbiome by comparing six tomato genotypes (*Solanum lycopersicum*) based on their traditional vs. commercial backgrounds. Using Illumina-based sequencing of the V6-V8 regions of 16S and ITS2 *rRNA* genes, we analyzed and compared the endophytic bacterial and fungal communities in stems to understand how microbiota can differ and be altered in plant genotypes and the relation to human manipulation. Our results reflect that traditional genotypes harbor significantly more exclusive microbial taxa and a broader phylogenetic background than the commercial ones. Traditional genotypes were significantly richer in *Eurotiomycetes* and *Sordariomycetes* fungi, while *Lasiosphaeriaceae* was more prevalent in commercial genotypes. TH-30 exhibited the highest bacterial abundance, significantly more than commercial genotypes, particularly in *Actinomycetia, Bacteroidia*, and *Gammaproteobacteria*. Additionally, traditional genotypes had higher bacterial diversity, notably in orders like *Cytophagales, Xanthomonadales*, and *Burkholderiales*. Moreover, we performed an evaluation of the impact of a systemic fungicide (tebuconazole-dichlofluanide) to simulate a common agronomic practice and determined that a single fungicide treatment altered the stem endophytic microbiota. Control plants had a higher prevalence of fungal orders *Pleosporales, Helotiales*, and *Glomerellales*, while treated plants were dominated by *Sordariomycetes* and *Laboulbeniomycetes*. Fungal community diversity significantly decreased, but no significant impact was observed on bacterial diversity. Our study provides evidence that the background of the tomato variety impacts the fungal and bacterial stem endophytes. Furthermore, these findings suggest the potential benefits of using of traditional genotypes as a source of novel beneficial microbiota that may prove highly valuable in unpredicted challenges and the advancement in sustainable agriculture.

## 1 Introduction

The plant microbiome has gathered considerable attention within the scientific community for its role in plant growth and resistance to several stresses (Pieterse et al., 2014; Berg et al., 2017; Gouda et al., 2018). Multiple factors shape the plant-associated microbial communities and their dynamics, including plant species, soil, location, and plant development stage (Zachow et al., 2014; Compant et al., 2019; Brown et al., 2020; Ishida et al., 2022). The plant genotype is recognized as a key factor in determining the microbiome (Wang et al., 2016; da Costa et al., 2022; Malacrinò et al., 2023), and plants may have evolved mechanisms to attract, select, and maintain their microbiota (Vorholt et al., 2017).

Human influence can also alter the microbiota through the pressure to domesticate plants in modern agriculture (Zachow et al., 2014; Pérez-Jaramillo et al., 2017; Zheng et al., 2020). Commercial genotypes, bred for optimal production in specific environments, may have retained only the essential microorganisms or core microbiota for those conditions (Neu et al., 2021; Favela et al., 2022), potentially leading to the absence of microorganisms found in more varied environments (Magurran and Henderson, 2003). In addition, current agronomic practices commonly involve pesticide treatments and their effects on the rhizosphere and soil microbiome have been reported (Nettles et al., 2016; Caradonia et al., 2019; Vozniuk et al., 2019; Chen et al., 2021; Zhang et al., 2021). This raises the question of the value of microorganisms absent under modern agronomic practices and whether they could be key to plant adaptation in unexpected challenging environments (Gera Hol et al., 2015; Ravanbakhsh et al., 2019).

Tomatoes (*Solanum lycopersicum)* are one of the worldwide main agricultural crops and several microbiome studies have evaluated the below-ground microbial communities (Poli et al., 2016; Chialva et al., 2018; Kwak et al., 2018; Gholizadeh et al., 2022; El-Debaiky and El-Sayed, 2023). However, research on aerial tissue microbiota is more limited (Ottesen et al., 2013; Runge et al., 2023) and studies on endophytic microbiota are even scarcer. In addition, most of the studies on genotype influence focus on commercial cultivars or wild tomato ancestors (French et al., 2020; Runge et al., 2023). Traditional cultivars, or landraces, represent a genetic middle ground between wild species, which exhibit low productivity and suboptimal quality for human consumption, and commercial cultivars that endure constant selection and agronomic treatments. These traditional cultivar plants undergo minimal selection pressure and are cultivated in small fields using traditional methods, including open pollination and fewer agronomic treatments (Ficiciyan et al., 2018). As a result, traditional tomatoes may harbor a unique and potentially untapped microbial reservoir that could differ significantly from the microbiota found in commercial cultivars. The significance of studying traditional tomatoes lies in their potential to contribute to agricultural biodiversity and sustainability. By understanding the microbial communities associated with these genotypes, we can explore new ways for improving crop resilience, disease resistance, and productivity through microbiome management. This could lead to the development of more sustainable agricultural practices that use the natural microbiota of traditional cultivars.

In this study, we analyze the endophytic stem microbiota of several tomato genotypes (*Solanum lycopersicum*), which objective is identifying and comparing the bacterial and fungal communities within the stems of both traditional and modern commercial genotypes. This is conduced through amplicon sequencing, which is a particularly effective method for exploring and profiling diversity and composition of the microbial communities. By contrasting the potential distinctions caused by genotype background, we aim to uncover novel insights into the role of traditional cultivars in shaping plant-associated microbiomes and their implications for crop improvement. The study of stem microbiota reduces the number of taxa bound to environmental factors and likely transmitted to the plant from the soil and air (Bulgarelli et al., 2013). While this could potentially be achieved by studying seed microbiota, which has been reported to harbor the core microbiota (Frank et al., 2017; Zhang et al., 2021), the use of developed plants also served to demonstrate the effects of a fungicide treatment. To explore the potential influence of a common crop management practice on the endophytic communities, we assessed the impact of systemic fungicides tebuconazole and dichlofluanide. Dichlofluanide is effective against a broad spectrum of diseases such as rust, black spot, *Botrytis cinerea*, and mildew by inhibiting spore germination. Tebuconazole, on the other hand, targets several pathogens including bunt, smut, net blotch, and powdery mildew by disrupting membranes and inhibiting sterol synthesis.

Our hypothesis asserts that traditional tomato plants, subjected to less selection and agronomic pressure, would harbor a more diverse microbiota compared to their cultivated counterparts. Similarly, we expected the treatment with fungicides would reduce the endophytic microbial diversity. The results are expected to contribute to the knowledge regarding the tomato microbiome, particularly in the context of endophytic stem microbiota, a relatively underexplored research area.

## 2 Materials and methods

### 2.1 Plant material

Six tomato (*Solanum lycopersicum* Mill.) genotypes were used in the study. Four tomato landraces from Spain (ADX2), Greece (TH-30), Israel (ISR-10), and France (MO-10) were selected alongside the widely used commercial cultivars Moneymaker (MM) and Ailsa Craig (AIL).

The seeds that were used to produce the plant material for this study were obtained from the Institute for the Conservation and Improvement of the Valencian Agrodiversity (COMAV), Polytechnic University of Valencia, Spain. They were kept in aseptic conditions, germinated and cultivated individually in pots containing 12 g of vermiculite as substrate in a growth chamber. The growth environment was controlled within the growth chamber (16-h photoperiod, 26°C:17°C day:night, 80% humidity). These measures were taken to minimize the effects of the environmental factors encountered typically in open-field conditions.

Plantlets were watered twice a week with Hoagland nutritive solution over 4 weeks, reaching the 5^th^ leaf stage. At that stage, a fungicide treatment was applied to two traditional varieties (ADX2, TH-30) and two commercial ones (AIL, MM). These specific genotypes were selected to ensure a comparison among plants of similar size. Five plants of each genotype were randomly selected for spray treatment with combined systemic fungicides (Tebuconazole 10% w/w + dichlofluanide 40% w/w) at a dosage of 5 mL per plant of a 0.25% solution, adhering to the manufacturer's recommendations (Folicur, Bayer). The remaining plants were mock-treated with distilled water. All plant material was collected 48 h post-fungicide treatment. This timeframe was chosen due to noticeable visual distinctions between treated and control plants, as treated plants exhibited reduced growth.

Sample preparation followed the methodological structure described by Sun et al. (2020). Stem tissue between 1^st^ and 4^th^ true leaves was cut and 1 cm long fragments were obtained from each internodal stem segment. These fragments were surface sterilized by immersion in a 4% bleach solution for 1 min, followed by 70% ethanol for 3 min and rinsed with sterilized distilled water.

Afterwards, 200 mg of each sample was transferred to Eppendorf tubes. These samples were used for DNA extraction, employing the CTAB method (Tamari et al., 2013) with DMSO to enhance strand separation. ITS4 (TCCTCCGCTTATTGATATGC) and ITS86 (GTGAATCATCGAATCTTTGAA) primers (Op De Beeck et al., 2014), with a melting point of 60°C, were used for fungal DNA amplification targeting the ITS2c region. For bacterial identification, 16S rDNA primers B969F (ACGCGHNRAACCTTACC) and BA1406R (ACGGGCRGTGWGTRCAA) (Walters et al., 2016) targeting the region V6–V8 were used, with a melting point of 62°C. Detailed procedures for this step are available in the full protocol (Comeau et al., 2017). Subsequently, agarose gel electrophoresis was performed to verify the DNA integrity of PCR samples.

For comparisons between commercial and traditional varieties, 10 plants per variety were used (10 × 6 varieties = 60 plants total). Comparisons between fungicide-treated and control plants involved 20 plants per condition with 5 plants per variety (5 × 4 varieties × 2 conditions = 40 plants in total). In addition, 150 mg of tissue sample was preserved in Eppendorf tubes and stored at −80°C for potential future use.

### 2.2 Amplicon sequencing

To perform sequencing of amplicons, samples were sent to the Integrated Microbiome Resource (IMR) at Morgan Langille Lab from the Department of Pharmacology at Dalhousie University (Canada). The protocols for the amplicon sequencing, barcoding adaptors and PCR conditions were described previously by Comeau et al. (2017). The amplicon fragments were PCR-amplified from the DNA in duplicate using separate template dilutions (1:1 & 1:10) using the Phusion™ High-Fidelity DNA Polymerase (Thermo Scientific™). A single round of PCR was done using fusion primers that include Illumina adaptors, indices and specific regions, targeting the ITS2 region for fungi analysis and the V6–V8 region of 16S for bacterial analysis. The PCR reactions from the same samples were pooled in one plate, then cleaned up and normalized using the high-throughput Just-a-Plate 96-well Normalization Kit (Charm Biotech™). All samples were then pooled to make one library which was quantified fluorometrically before sequencing. The amplicon samples were run on Illumina MiSeq using 300 + 300 bp paired-end V3 chemistry which allows overlap and stitching together of paired amplicon reads into one full-length read of higher quality.

### 2.3 Bioinformatics analysis

Forward and reverse reads were imported and demultiplexed using the QIIME2 platform (version 2020.2; https://qiime2.org). Sequence quality control and feature table construction were performed using *Deblur workflow*, applying a trimming value of 250 as described by Amir et al. (2017). The bacterial samples were analyzed under the *Deblur denoise-16S* build-in protocol, whereas quality control and construction for ITS were performed with UNITE Version 8.2 (https://unite.ut.ee/) as a reference. The taxonomic composition of the samples was assessed with the classifier feature *classify-consensus-blast* using UNITE version 8.2 as a reference database for ITS and *Greengenes 2* 99% operational taxonomic units (OTUs) for 16S with a resolution of 99% (McDonald et al., 2012). Since the DNA was extracted from plant tissue, the presence of plant mitochondria and chloroplast might arise. Hence, a taxa filtration to exclude these terms was performed following the protocol in Comeau et al. (2017). For diversity analysis, the table of frequencies was rarefied (subsampled) according to the values obtained from the deblur workflow for ITS and 16S at sequencing depths of 1,000 and 5,000 respectively. Afterwards, the obtained results were subjected to statistical analysis performed by different software packages. α-diversity metrics were achieved through the QIIME2 package. The resulting data was imported to R statistical software (R version 4.0.2) using the qiime2R package. Principal Coordinates Analysis (PCoA), taxabars, hierarchical clusters, and diversity plots were executed with the *Microbiotaprocess* ver. 1.2.2 and the *Vegan* ver. 2.6-2 packages. Shannon graphs were made using the *Tidyverse* package ver. 1.3.1. The abundance of taxa was calculated using the *Phyloseq* package ver. 1.34.0 and phylloclades were calculated with the *Coin* ver. 1.4-1 package.

A total of 3,823,640 reads for ITS sequences and 2,709,671 reads for 16S sequences were retrieved from the analyzed plant samples under control conditions after filtering for mitochondria and chloroplast (0.1%). Among these, 237 OTUs belonged to fungi, of which 232 aligned successfully with the UNITE reference database. All 343 bacterial OTUs aligned successfully with the Greengenes 2 database.

## 3 Results

### 3.1 Distribution and overall comparison of the endophytic OTUs

**Figure 1:**
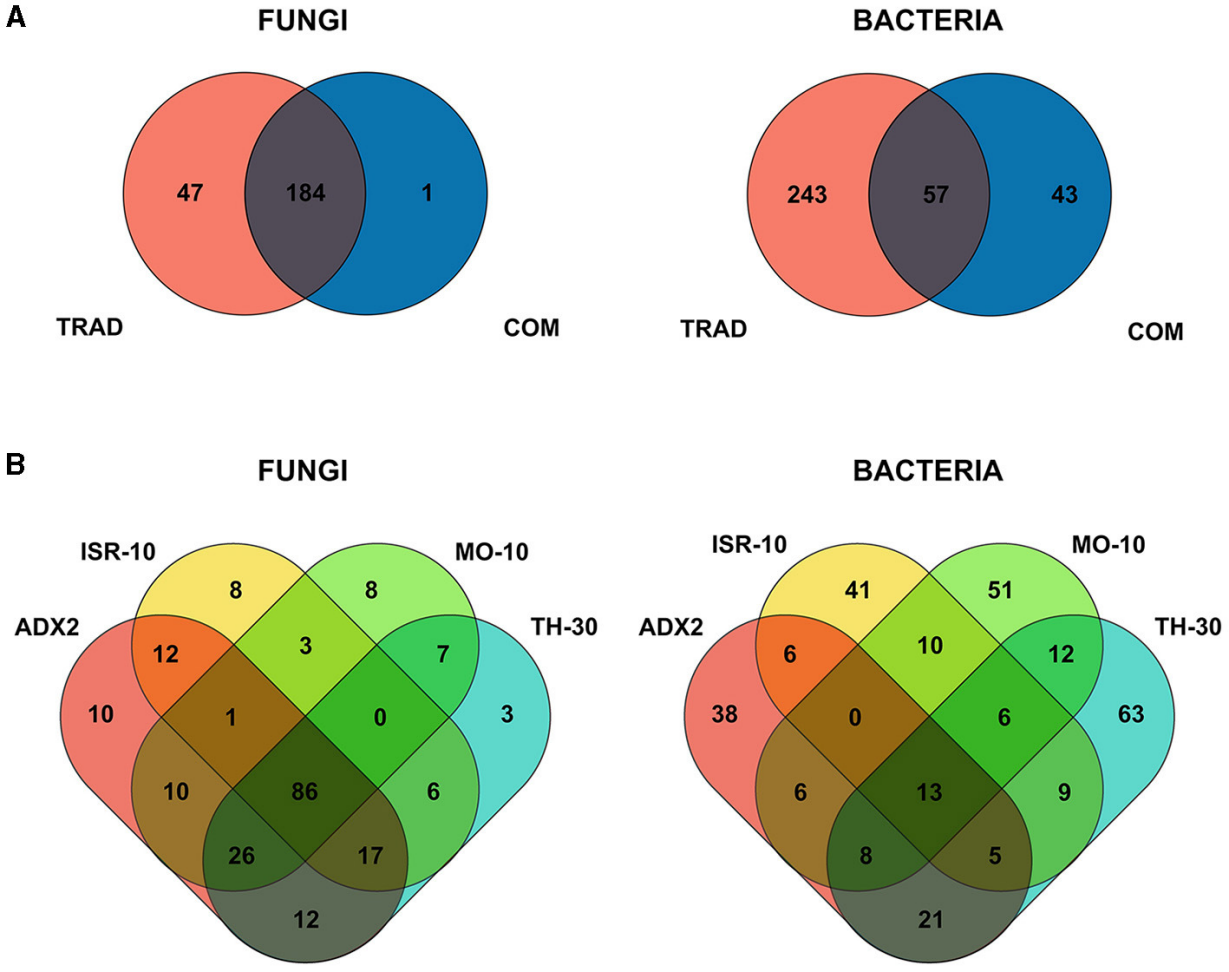
Venn Diagrams of the different microbial operational taxonomic units (OTUs) of four traditional varieties (ADX2, TH-30, ISR-10, MO-10) and two commercial cultivars (AIL, MM) of *S. lycopersicum*. Ellipsoidal areas represent the study groups, with common OTUs represented in overlapped areas. **(A)** Overall comparison between OTUs from traditional and commercial genotypes for fungal and bacterial endophytic communities. **(B)** Common and exclusive OTUs between traditional varieties for fungal and bacterial taxa.

Venn diagrams were generated to compare the overall OTUs between traditional and commercial genotypes of tomato (Figure 1A). One hundred and eighty four different fungal OTUs were shared by both traditional and commercial genotypes while 47 OTUs were exclusive of traditional tomato genotypes (Supplementary Table 1). Commercial genotypes held only one exclusive OTU identified as an uncultured Ascomycota. For bacteria, 57 OTUs were shared, 243 exclusive to traditional genotypes (Supplementary Table 2), and 43 exclusive to commercial genotypes.

The distribution among traditional genotypes (Figure 1B) showed a common core of 86 fungal OTUs. The number of exclusive OTUs was higher in ADX2 (10), ISR-10 (8), MO-10 (8), and lower in TH-30 (3). ADX2 harbored the highest number of total OTUs (188).

On the other hand, only 13 bacterial OTUs were common among the traditional genotypes, and each genotype harbored several exclusive OTUs. TH-30 possessed the highest number (63), followed by MO-10 (51), ISR-10 (41), and ADX2 (38). TH-30 harbored the highest number of total OTUs (137).

**Figure 2:**
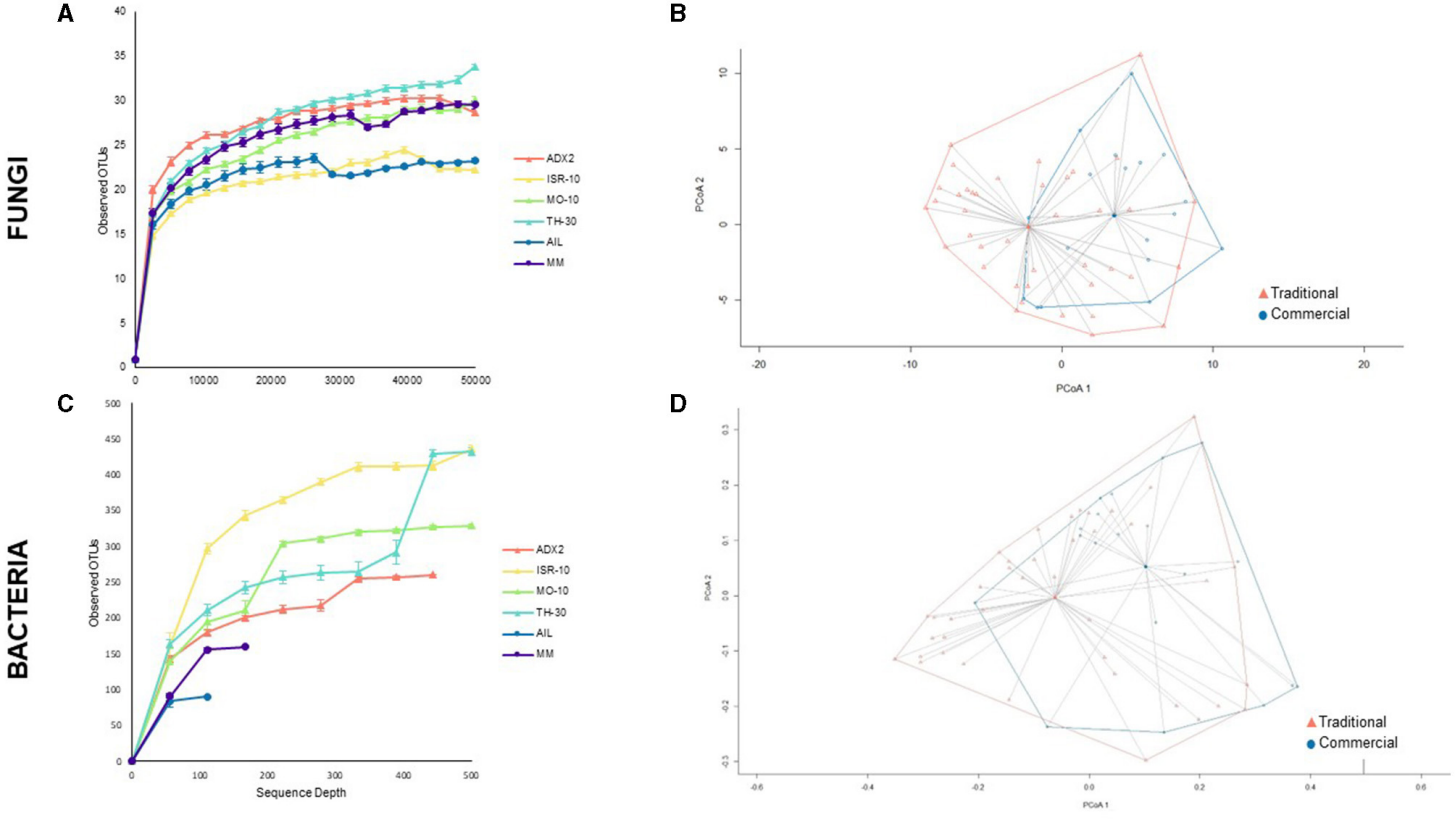
Diversity of fungal **(A, B)** and bacterial **(C, D)** endophytes according to genotype or genotype origin. **(A, C)** α-diversity of microbial communities for each tomato genotype shown by rarefaction curves based on number of observed OTUs, represented as mean ± standard deviation **(B, D)** Principal Coordinates Analysis (PCoA) plot of microbial communities between traditional and commercial genotypes, with distance matrices based on the Bray-Curtis dissimilarity index and highlighting the clustering of samples according to genotype origin.

The overall α-diversity, as measured by observed OTUs, was represented in rarefaction curves (Figures 2A–C). Significant differences in fungal and bacterial diversity were observed, although no clear distinction between the traditional and commercial groups was found for fungi. In addition, the differences between the genotypes were analyzed by several α-diversity indexes (Supplementary Figures 1, 2).

Bray-Curtis dissimilarity was used to study β-diversity (Figures 2B–D). PCoA plots were generated by clustering samples of each background (traditional or commercial) based on unweighted unifrac calculations, as weighted unifrac did not contribute in defining results. The distance between the centroids of the clusters was 5.17 for fungi and 0.618 for bacteria which indicates a greater dissimilarity in fungal communities between traditional and commercial groups. This distance was mainly attributed to the first component. To assess the statistical significance of group clustering, Permutational Multivariate Analysis of Variance (PERMANOVA) tests were conducted. The difference was found to be statistically more significant for fungal samples (Pr > F 0.001), than bacterial ones (Pr > F 0.004).

### 3.2 Composition of the endophytic communities

**Figure 3:**
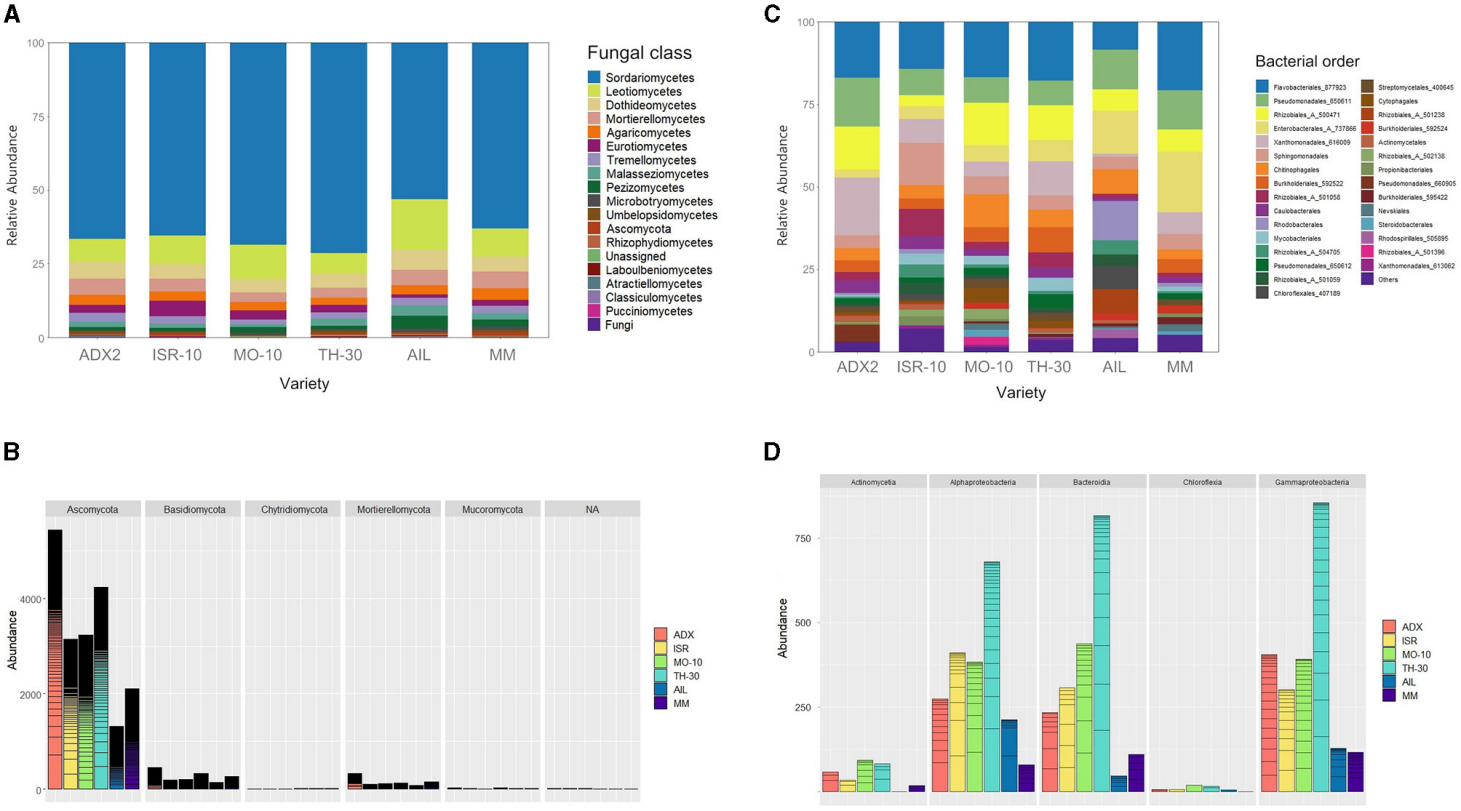
Abundance and structure of fungal and bacterial endophytic communities within the host for each studied tomato genotype. **(A)** Relative taxonomic composition and structure of fungi expressed at class rank. **(B)** Distribution and abundance of main taxonomic phyla in fungal communities: *Ascomycota, Basidiomycota, Chytridiomycota, Mortierellomycota*, and *Mucoromycota*. **(C)** Relative taxonomic composition and structure of bacteria expressed at order rank. **(D)** Distribution and abundance of main taxonomic classes in bacterial communities*: Actinomycetia, Alphaproteobacteria, Bacteroidia, Chloroflexia*, and *Gammaproteobacteria*.

The composition and abundance of the microbial communities were examined and depicted in bar plot graphics (Figure 3) at a taxonomic level that ensured item distinguishability and clarity.

The structure of fungal OTUs at the class level (Figure 3A) was consistent across all tomato genotypes. The fungal communities were dominated by *Sordariomycetes* (>50%), followed by other ascomycetes from the classes Leotiomycetes (5%−15%) and Dothideomycetes (4%−6%). The Basidiomycota phylum was primarily represented by taxa belonging to the classes Agaricomycetes (2%−4%), Tremellomycetes (1%−3%), and Malasseziomycetes (1%−4%). Other phyla, such as Chytridiomycota and Mucoromycota, exhibited low prevalence (<1%).

The Ascomycota prevalence was also evident in absolute abundance (Figure 3B). The abundance of subgroups appeared as horizontal lines in the bars though this did not convey further relevant information. The statistical differences among genotypes were analyzed using Fisher's Least Significant Difference (LSD) test, with the significance set at *p* < 0.05 (Supplementary Table 3). Commercial AIL hosted significantly fewer ascomycotes than ADX2 and TH-30, and commercial MM had significantly fewer than ADX2. No significant distinctions were found in other fungal phyla based on the genotype background. Although significant distinctions among traditional genotypes were identified, some could not be distinguished from the commercial genotype MM.

Regarding the composition of bacterial endophytic communities (Figure 3C), a similar distribution was observed across all genotypes except for AIL. A significant portion was covered by the orders Flavobacteriales (9%−22%), Sphingobacteriales (4%−14%), and Chitinophagales (4%−10%), contributing to the prominence of the Bacteroidia class. Gammaproteobacteria was also prevalent, including the orders Pseudomonadales (11%−17%), Enterobacterales (2%−19%), and Xanthomonadales (1%−18%), while the order Rhizobiales (14%−18%) was the primary contributor to the abundance of Alphaproteobacteria. The AIL genotype was notable for its higher presence of Rhodobacteriales and a subgroup of Rhizobiales (504705), along with a relative decrease in Flavobacteriales compared to other genotypes.

The traditional genotype TH-30 exhibited the highest bacterial abundance (Figure 3D), which was statistically distinct from the commercial genotypes (*p* < 0.05), specifically for the bacterial classes of Actinomycetia, Bacteroidia, and Gammaproteobacteria.

**Figure 4:**
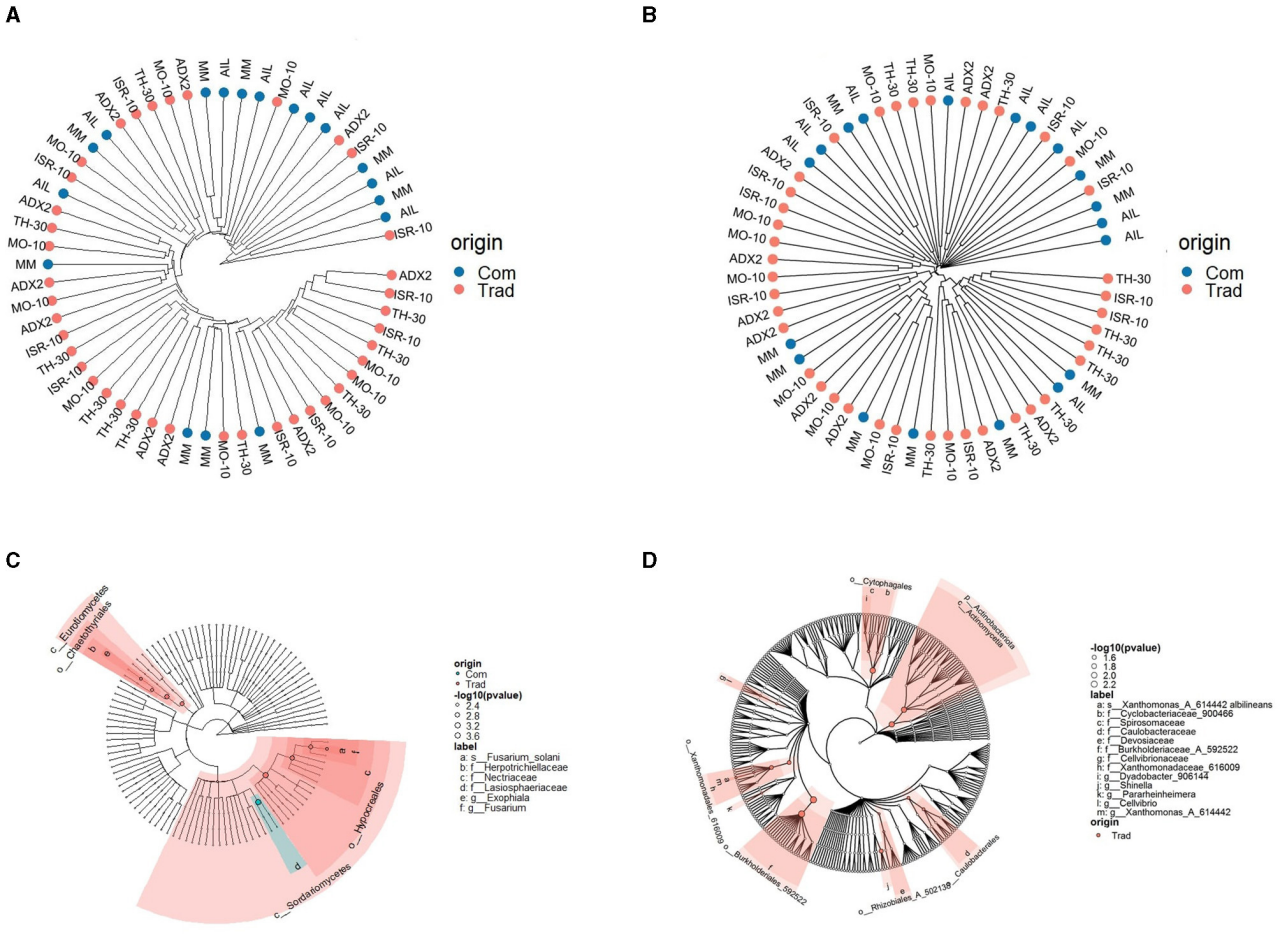
Cladogram layout for the stem microbiota in *S. lycopersicum* with the distinction between traditional and commercial genotypes. **(A)** Euclidean hierarchical cluster of fungal samples. **(B)** Euclidean hierarchical cluster of bacterial samples. **(C)** Phylogenetic cladogram of fungal OTUs, with a highlighted area representing greater density for OTUs from a particular tomato origin (commercial or traditional). **(D)** Phylogenetic cladograms of bacterial OTUs, with a highlighted area representing greater density for OTUs from a particular tomato origin (commercial or traditional).

The phylogenetic structure of the microbial communities was represented in cladograms (Figure 4). The hierarchical cluster analysis (HCA) of the samples (Figures 4A, B) indicated a distinction that was particularly noticeable in fungal clusters, where AIL samples clustered closely, indicating a narrower phylogenetic composition and variation than other genotypes. Phylogenetic cladograms also showed differences based on genotype background (Figures 4C, D). The highlighted areas indicated a significantly higher density of microbial reads from either the traditional or commercial genotypes, as determined by the Kruskal-Wallis test. Traditional genotypes were significantly richer in fungi from the Eurotiomycetes and *Sordariomycetes* classes, although the family Lasiosphaeriaceae was significantly more prevalent in commercial genotypes. Traditional genotypes also had higher presence of the bacterial orders Cytophagales, Xanthomonadales, Burkholderiales, Rhizobiales, and Caulobacteriales.

### 3.3 Variation of the endophytic communities under fungicide treatment

**Figure 5:**
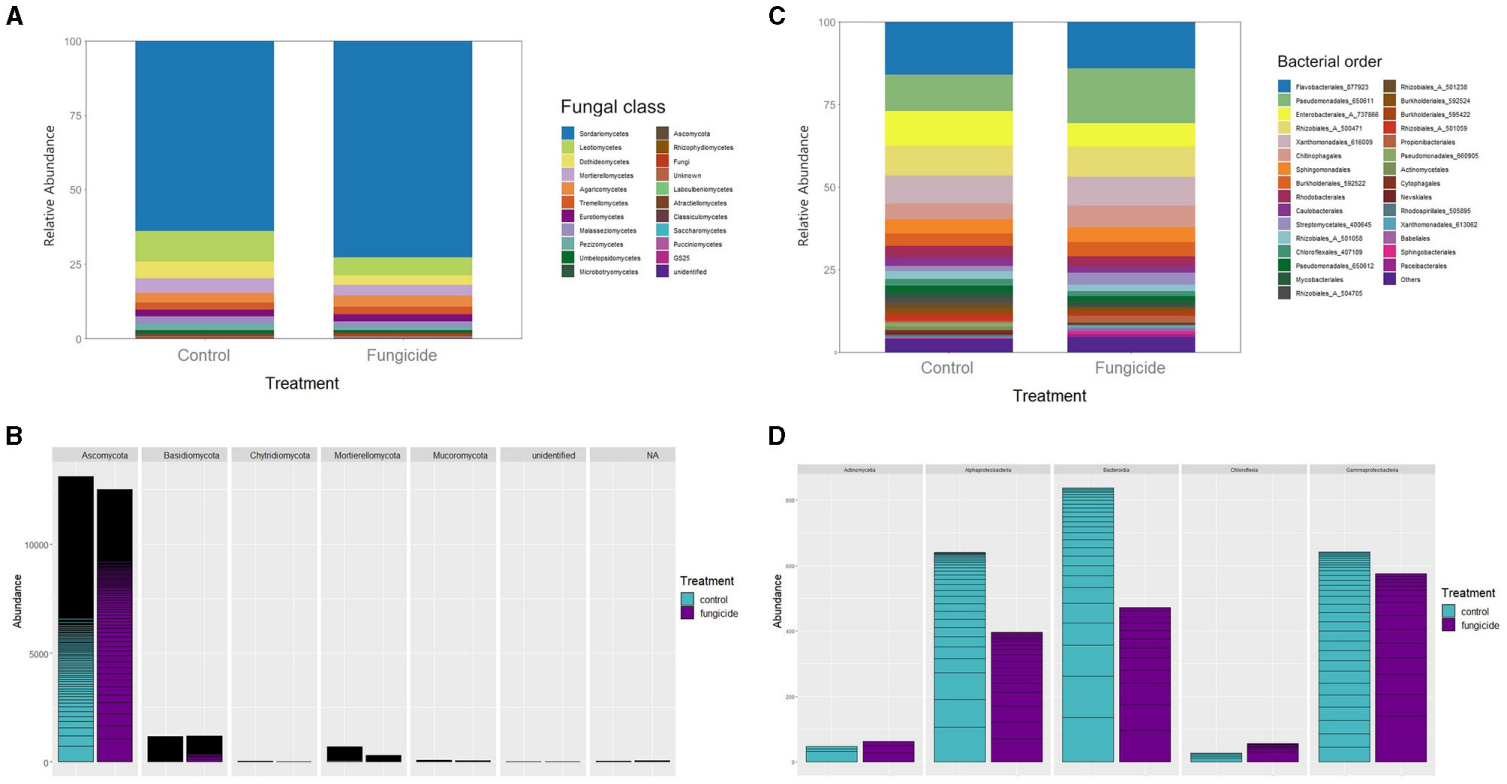
Abundance and structure of fungal and bacterial endophytic communities in *S. lycopersicum* for control (mock-treated) and fungicide-treated plants (ADX2, TH-30, AIL, and MM genotypes). **(A)** Relative taxonomic composition and structure of fungi expressed at class rank. **(B)** Distribution and abundance of main taxonomic phyla in fungal communities: *Ascomycota, Basidiomycota, Chytridiomycota, Mortierellomycota*, and *Mucoromycota*. **(C)** Relative taxonomic composition and structure of bacteria expressed at order rank. **(D)** Distribution and abundance of main taxonomic classes in bacterial communities*: Actinomycetia, Alphaproteobacteria, Bacteroidia, Chloroflexia*, and *Gammaproteobacteria*.

The impact of the systemic fungicides dichlofluanid and tebuconazole, applied via spray on the tomato plants, was not apparent on the structure of endophytic microbiota (Figures 5A–C) (*p* < 0.05). However, the abundance of some fungal and bacterial taxa did significantly decrease for TH-30 (Figures 5B–D, Supplementary Table 4). There were some instances where fungicide treatment resulted in an increased relative abundance of specific taxa. This increase was particularly noticeable in bacterial taxa for AIL and TH-30 (Supplementary Table 5) and in fungal taxa for AIL and MM (Supplementary Table 6). Specifically, AIL showed a notable increase in *Hypocreales*, while *Helotiales* and *Pleosporales* were the most increased orders in MM.

**Figure 6:**
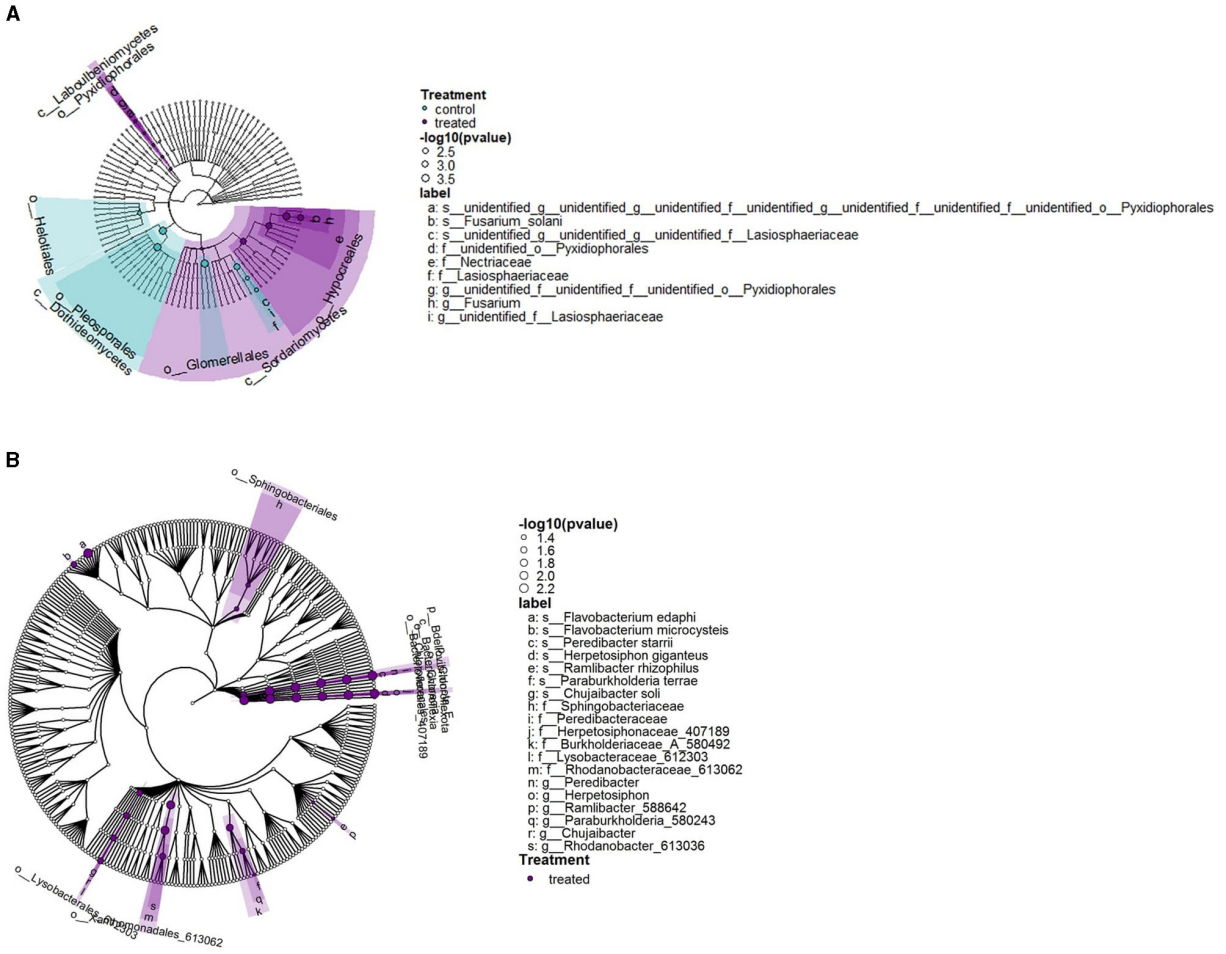
Cladogram layout for the stem microbiota in *S. lycopersicum* with distinction between control (mock-treated) and fungicide-treated plants**. (A)** Phylogenetic cladogram of fungal OTUs. The highlighted area represents greater density for OTUs from one of the conditions (mock-treated, fungicide-treated). **(B)** Phylogenetic cladogram of bacterial OTUs. The highlighted area represents greater density for OTUs from one of the conditions (mock-treated, fungicide-treated).

The phylogenetic distribution of OTUs highlighted the main differences between fungicide-treated and control plants (Figure 6). In control plants, the fungal orders *Pleosporales, Helotiales*, and *Glomerellales* predominated, whereas treated plants were notable for their abundance of *Sordariomycetes* and *Laboulbeniomycetes* classes. Fungicide treatment also led to a higher incidence of specific bacterial families such as *Sphingobacteriaceae, Peredibacteraceae* (*Bacteriovoracaceae*), and *Rhodanobacteraceae*, with no prevalence in any clade or common ancestor.

**Figure 7:**
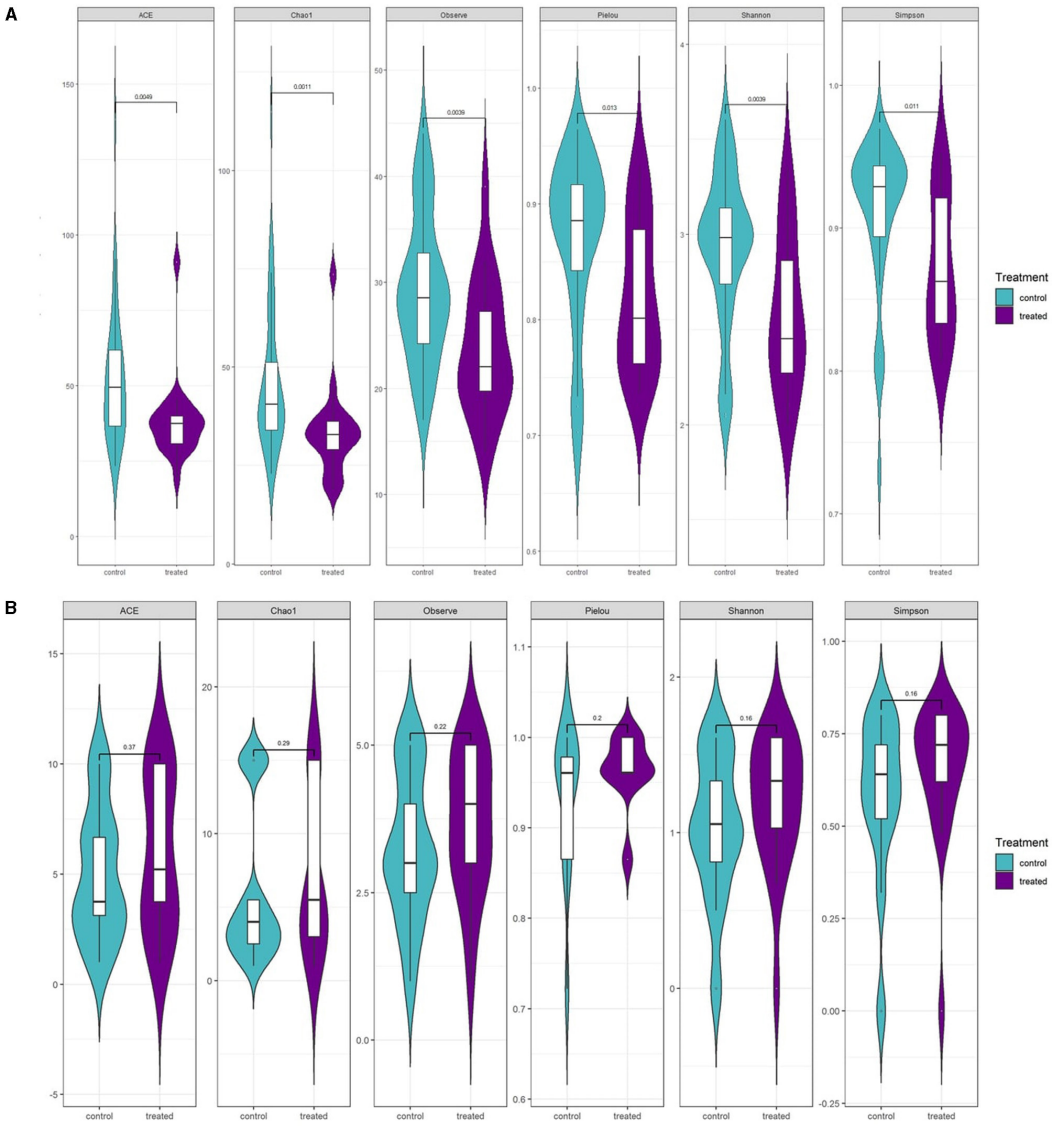
Estimated richness of control vs. fungicide-treated *S. lycopersicum* by several diversity tests for **(A)** fungal communities and **(B)** bacterial communities. The calculated diversity index corresponds to Observe, Chao1, ACE, Shannon, Simpson, and J test with a *P*-value based on the Wilcoxon test (*n* = 10).

In addition, several α-diversity indices revealed a significant reduction in fungal community diversity post-fungicide application (Figure 7) (*p* < 0.01). Treated plants showed lower fungal richness based on Chao1 and ACE tests and significantly reduced fungal diversity according to the Shannon test. Moreover, Pielou's Evenness Index indicated a higher degree of inequality among communities in treated plants. Conversely, fungicide treatment did not show any statistically significant impact on bacterial diversity.

## 4 Discussion

This study aimed to evaluate the endophytic stem microbiota of various tomato genotypes used for consumption, comparing their similarities and differences based on the genotype background (traditional vs. commercial). By focusing specifically on the bacterial and fungal endophytic communities in stems, this research seeks to expand the current understanding of the tomato microbiome, which is typically focused on the rhizosphere and bacterial populations. Additionally, it helped to delve into the impact of the aerial application of a combination of systemic fungicides on the endophytic communities of the plant.

Our study showed that in all the genotypes tested, *Sordariomycetes, Dothidiomycetes*, and *Leotimycetes* are the predominant classes. Interestingly, Manzotti et al. (2020) observed similar results in Castlemart variety, which exhibited a significantly higher proportion of *Sordariomycetes* compared to the wild type. This similar pattern may suggest that the predominant classes are able to colonize the whole plant. The connection between plant compartments has been previously found in other species (Wang et al., 2016; Cregger et al., 2018) and suggests a feasible resemblance of our results with the microbiota in other tissues of our study genotypes. On the other hand, Dong et al. (2021), analyzed endophytic communities of tomato “Zhongza 302,” and observed that *Sordariomycetes* and *Dothidiomycetes* were predominant in roots with a similar distribution, while *Dothidiomycetes* dominated in their stems (around 70%). This, reinforces the hypothesis that origin or development of the cultivar may highly influence the composition of the microbiome.

The bacterial communities identified in this study revealed a prevalence of *Gammaproteobacteria*. In this case, the results observed were different from those previous reported for root-focused investigations (French et al., 2020; Haq et al., 2021) and tomato leaf microbiome (Toju et al., 2019; Llontop et al., 2021). While *Bacteroidia* emerged as the dominant taxa in roots, *Sphingomonadales* were predominant in the leaves of grafted tomato. Interestingly, Zhang et al. (2022), observed that the differences in the management of the tomato plants could affect the composition of the plant microbiome in different compartments. These findings underscore the considerable variability in bacterial communities across different plant genotypes and compartments, which could stem from bacterial transmission dynamics (Wang et al., 2016), higher adaptation to varying conditions or sensitivity to stochastic processes (Taniguchi et al., 2023) or likely a combination of them.

Focusing on the differences in the stem endophytic microbiota among the genotypes in our study, several divergences were observed. While fungal OTUs presented a strong shared core across all genotypes, bacterial OTUs were more specific to each genotype. The variability in the microbiome among plant genotypes of the same species have been reported multiple times. Several studies have identified cultivar-specific microorganisms in various crops, including maize, sweet potato, wheat, pea, oat, and barley (Inceoglu et al., 2011; Fitzpatrick et al., 2018; Peiffer et al., 2013; Turner et al., 2013; Marques et al., 2014; Bulgarelli et al., 2015).

In our study, *Ascomycetous* fungi dominated across all the genotypes, which is consistent with their widespread presence in plants (Camarena-Pozos et al., 2021). Traditional genotypes exhibited a higher presence of *Herpotrichiellaceae* and *Nectriaceae* families, while commercial genotypes showed higher presence of *Lasiosphaeriaceae*. The results of fungal α- and β-diversity indicated an imbalance in fungal communities. Regarding bacteria, traditional genotypes exhibited a higher abundance of taxa from *Flavobacteriales*, whereas commercial genotypes showed a notable prevalence of *Enterobacterales* and *Rhodobacterales*. Bacterial α-diversity was significantly lower in commercial varieties, and β-diversity supported the existence of differences.

These findings suggest that the substantial difference in microbial composition between traditional and commercial varieties lies more in the bacterial endophytes than the fungal ones, manifesting higher differences in both abundance and diversity. We observed that lower bacterial abundance was mostly correlated with lower fungal abundance in the genotypes. It has been previously hypothesized that the presence of fungal hyphae may have an impact on the bacterial recruitment (Zhang et al., 2024). Notably, the commercial AIL and MM showed the lowest total microbial abundance and a narrower phylogenetic background, therefore reinforcing our main hypothesis regarding the distinction between commercial and traditional genotypes. In previous works comparing modern cultivars with wild tomato plants, Yu J. et al. (2023) observed that agricultural conditions such as phosphorus level can change the composition of plant microbiome, suggesting that domestication not only changed the genotype but also influenced the plant microbiome with the transition from native habitats to agricultural soils.

These results suggests that the continuous pressure on commercial tomato cultivation does not alter the overall structure of fungal communities but may reduce the endophytic diversity and induce significant changes at low taxonomic level. The disparities reflected here need further investigation to clarify whether it is entirely attributed to human manipulation or rather an accumulation of genotypical differences over time.

On the other hand, pesticides are treatments that are typically applied directly to soil or by spray to foliage, thus having a greater impact on the microbial community on the rhizosphere and soil microbiome (Nettles et al., 2016; Vozniuk et al., 2019; Zhang et al., 2021), and on the phyllosphere (Chen et al., 2021). However, the effects of these chemical treatments on endophytic communities are not addressed as much. These could similarly affect the stem endophytic communities, potentially influencing plant physiology or even posing risk for consumption (Yu Z. et al., 2023).

Our results revealed that tebuconazole and dichlofluanide disrupted the balance of the tomato endophytic microbiota leading to a reduction of certain community members while others persist. This disruption may not cause direct negative effects on plant performance, though the persistence of these treatments might alter the microbial communities significantly. Although most of these communities appear to be environmentally bound with no notable pathogenic tendencies, our study revealed a significant presence of *Fusarium solani* in the fungicide-treated plants. This observation may indicate the persistence of this pathogen and its potential negative impact on the plants. On the other hand, the use of fungicides could translate into higher bacteria colonization by reducing competition (Lu et al., 2019), observed in a significantly higher presence of some families such as *Sphingobacteriacea, Peredibacteraceae*, and *Herpetosiphonaceae*. Nevertheless, the application of the fungicides did not concur in significant changes in the bacterial diversity. As non-target communities, other studies focused on phyllosphere and soil also observed that no significant changes happened in the bacterial communities (Perazzolli et al., 2014; Prudnikova et al., 2020; Wu et al., 2023). In addition, the use at recommended rates of tebuconazole might not significantly change bacterial communities, as previously observed in soil by Volova et al. (2017).

Despite of that, certain communities may exhibit greater resistance against fungicide treatments. It is currently known that some bacteria can degrade pesticides which gives them an advantage against other microorganisms (Satapute and Kaliwal, 2016; Alexandrino et al., 2020). Han et al. (2021) observed that in tebuconazole-treated soils bacteria that can degrade this fungicide (*Methylobacterium, Burkholderia, Hyphomicrobium*, and *Dermacoccus*) increase their activity. In our case, we found the presence of OTUs belonging to these genera (except for *Dermacoccus*) and it could be plausible that these communities unbalanced the composition of the microbiome. Another relevant point to take into account is bacteria sensitivity. A previous study on grapevine observed that bacterial communities displayed varying sensitivity to different fungicides (Andreolli et al., 2023).

As a particular instance, the traditional genotype TH-30 did exhibit notable changes that were not shared with the other genotypes. This included a significant reduction in the abundance of Ascomycota, *Alphaproteobacteria, Bacteroidia*, and *Gammaproteobacteria*. We hypothesize that this increased fluctuation observed in the TH-30 endophytic microbiota may be attributed to a delicate microbial balance, potentially contributing to its high susceptibility under stressful conditions, as noted by Fernández-Crespo et al. (2022).

Ultimately, we believe that non-target effects must be considered during the formulation and application of pesticides. Our study indicates that even a single application of fungicide can induce changes in the endophytic communities, which may lead to imbalances. If the long-term effects of repeated applications lead to cumulative changes over adaptation (Sim et al., 2023), this could result in noticeable distinctions between genotypes subject to continuous treatment and those that are not. However, this aspect requires further investigation.

## 5 Conclusions

The relationship between plant genotype and microbiome has been previously documented in several crop species (Zachow et al., 2014; Pérez-Jaramillo et al., 2017; Zheng et al., 2020). However, the characterization of tomato endophytic communities remains fairly unexplored. This study represents a first effort to compare stem microbiota among tomato genotypes from both traditional and commercial background. Our results indicate that traditional tomato genotypes, subjected to less manipulation, host several exclusive taxa that are absent in their commercial counterparts. Furthermore, the PERMANOVA analysis revealed that their microbial communities were distinguished from those of commercial genotypes. This suggests that traditional genotypes may serve as a richer reservoir of potentially novel endophytes, which hold promising alternatives for enhancing plant health in sustainable agriculture. Additionally, we observed that a systemic fungicide treatment could change the stem endophytic microbiota. Although the impact was not evident in the microbial community structure, the treatment induced changes at a low taxonomic level and led to a decrease in fungal α-diversity. This supports the notion that chemical treatments may influence endophytic microbiota and contribute to the differences over time found between traditional and commercial genotypes. We believe that these findings will contribute to enhancing our understanding of the tomato microbiome, as this will be a valuable resource for the demanding future of agronomy.

## Data Availability

The datasets presented in this study can be found in online repositories. The names of the repository/repositories and accession number(s) can be found below: https://osf.io/smvra/; doi: 10.17605/OSF.IO/SMVRA.
