# Supplementary material for: Exploring the impact of plant genotype and fungicide treatment on endophytic communities in tomato stems
Source: Front Microbiol. 2024 Sep 25;15:1453699. doi: 10.3389/fmicb.2024.1453699 (PMC11469548; doi:10.3389/fmicb.2024.1453699)
Supplement: Supplementary file 2 [file Data_Sheet_1.docx]

Supplementary Material

**Supplementary Table 1.** Unique fungal taxa found in traditional genotypes of tomato (ADX2, ISR-10, MO-10, TH-30) and absent from the commercial genotypes.

| Class | Order | Species | Consensus |
| --- | --- | --- | --- |
| Dothideomycetes | Pleosporales | Unidentified | 0.89 |
| Tremellomycetes | Filobasidiales | *Solicoccozyma sp.* | 0.56 |
| Sordariomycetes | Sordariales | Unidentified | 0.90 |
| Leotiomycetes | NA | Unidentified | 0.80 |
| Sordariomycetes | Sordariales | *Conlarium sp.* | 0.70 |
| Tremellomycetes | Cystofilobasidiales | Unidentified | 0.80 |
| Sordariomycetes | Sordariales | Unidentified | 0.60 |
| Pucciniomycetes | Platygloeales | Unidentified | 0.60 |
| Sordariomycetes | Sordariales | *Conlarium sp.* | 0.78 |
| Dothideomycetes | Pleosporales | Unidentified | 0.80 |
| Eurotiomycetes | Chaetothyriales | Unidentified | 0.70 |
| Laboulbeniomycetes | Pyxidiophorales | unidentified | 1.00 |
| Sordariomycetes | Hypocreales | Unidentified | 0.90 |
| Eurotiomycetes | Chaetothyriales | *Exophiala sp.* | 0.60 |
| Leotiomycetes | Helotiales | Unidentified | 0.90 |
| Sordariomycetes | Sordariales | Unidentified | 0.90 |
| NA | NA | Unidentified | 1.00 |
| Tremellomycetes | Holtermanniales | *Holtermanniella sp.* | 0.56 |
| Sordariomycetes | Microascales | *Cephalotrichum sp.* | 0.60 |
| Eurotiomycetes | Eurotiales | *Talaromyces sp.* | 1.00 |
| Sordariomycetes | Myrmecridiales | Unidentified | 0.71 |
| Sordariomycetes | Sordariales | *Conlarium sp.* | 0.70 |
| Pezizomycetes | Pezizales | Unidentified | 1.00 |
| Atractiellomycetes | Atractiellales | Unidentified | 0.67 |
| Leotiomycetes | Helotiales | *Tetracladium sp.* | 0.60 |
| Leotiomycetes | Helotiales | Unidentified | 0.80 |
| Sordariomycetes | Hypocreales | *Trichoderma sp.* | 1.00 |
| Sordariomycetes | Hypocreales | Unidentified | 0.80 |
| Sordariomycetes | Hypocreales | Unidentified | 1.00 |
| Tremellomycetes | Tremellales | *Vishniacozyma sp.* | 0.60 |
| Sordariomycetes | Sordariales | Unidentified | 0.60 |
| Sordariomycetes | Sordariales | Unidentified | 0.80 |
| Sordariomycetes | Sordariales | *Chaetomium sp.* | 0.60 |
| Agaricomycetes | Polyporales | *Ganoderma sp.* | 1.00 |
| Sordariomycetes | Hypocreales | Unidentified | 1.00 |
| Eurotiomycetes | Eurotiales | *Talaromyces sp.* | 0.90 |
| Mortierellomycetes | Mortierellales | *Mortierella sp.* | 0.90 |
| Malasseziomycetes | Malasseziales | *Malassezia sp.* | 0.86 |
| Sordariomycetes | Sordariales | Unidentified | 0.90 |
| Dothideomycetes | Pleosporales | Unidentified | 0.63 |
| Leotiomycetes | NA | Unidentified | 0.80 |
| Leotiomycetes | Thelebolales | *Pseudeurotium sp.* | 0.60 |
| Agaricomycetes | Agaricales | *Psathyrella sp.* | 1.00 |
| Leotiomycetes | Helotiales | *Neobulgaria sp.* | 0.60 |
| Leotiomycetes | Helotiales | *Oidiodendron sp.* | 1.00 |
| Sordariomycetes | Sordariales | Unidentified | 0.80 |
| Sordariomycetes | Glomerellales | Unidentified | 0.60 |

**Supplementary Table 2.** Unique bacterial taxa found in traditional genotypes of tomato (ADX2, ISR-10, MO-10, TH-30) and absent from the commercial genotypes.

| Class | Order | Species | Consensus |
| --- | --- | --- | --- |
| Alphaproteobacteria | Rhizobiales | *Agrobacterium sp.* | 1.0 |
| Gammaproteobacteria | Enterobacterales | *Pectobacterium cacticida* | 1.0 |
| Gammaproteobacteria | Burkholderiales | *Methylophilus methylotrophus* | 1.0 |
| Gammaproteobacteria | Burkholderiales | *Acidovorax soli* | 1.0 |
| Gammaproteobacteria | Burkholderiales | *Methylophilus rhizosphaerae* | 1.0 |
| Bacteroidia | Chitinophagales | *Sediminibacterium sp.* | 1.0 |
| Bacteroidia | Chitinophagales | *Edaphocola sp003852495* | 1.0 |
| Bacteroidia | Sphingobacteriales | *Mucilaginibacter sp.* | 1.0 |
| Actinomycetia | Streptomycetales | *Streptomyces kaempferi* | 1.0 |
| Alphaproteobacteria | Rhizobiales | *Rhizobium miluonense* | 1.0 |
| Gammaproteobacteria | Enterobacterales | *Pectobacterium aquaticum* | 1.0 |
| Alphaproteobacteria | Sphingomonadales | *Sphingopyxis indica* | 1.0 |
| Gammaproteobacteria | Burkholderiales | *Methylophilus sp000515275* | 1.0 |
| Bacteroidia | Flavobacteriales | *Flavobacterium edaphi* | 1.0 |
| Gammaproteobacteria | Xanthomonadales | *Lysobacter antibioticus* | 1.0 |
| Bacteroidia | Chitinophagales | *Terrimonas sp.* | 1.0 |
| Alphaproteobacteria | Rhizobiales | *Rhizobium tropici_500522* | 1.0 |
| Gammaproteobacteria | Burkholderiales | *Ideonella azotifigens* | 1.0 |
| Vampirovibrionia | N/A | *Unidentified* | 1.0 |
| Bacteroidia | Cytophagales | *Chryseolinea serpens* | 1.0 |
| Actinomycetia | Mycobacteriales | *Mycobacterium sp.* | 1.0 |
| Actinomycetia | Streptomycetales | *Streptomyces achromogenes* | 1.0 |
| Bacteroidia | Flavobacteriales | *Flavobacterium bizetiae* | 1.0 |
| Bacteroidia | Flavobacteriales | *Flavobacterium ummariense* | 1.0 |
| Gammaproteobacteria | Xanthomonadales | *Arenimonas sp.* | 1.0 |
| Bacteroidia | Chitinophagales | *Terrimonas sp.* | 1.0 |
| Actinomycetia | Actinomycetales | *Microbacterium arthrosphaerae* | 1.0 |
| Bacteroidia | Flavobacteriales | *Fluviicola kyonggii* | 1.0 |
| Bacteroidia | Cytophagales | *Chryseolinea sp.* | 1.0 |
| Gammaproteobacteria | Burkholderiales | *Neisseria subflava* | 1.0 |
| Bacteroidia | Flavobacteriales | *Moheibacter sediminis* | 1.0 |
| Gammaproteobacteria | Burkholderiales | *Leptothrix discophora* | 1.0 |
| Bacteroidia | Flavobacteriales | *Chryseobacterium shandongense* | 1.0 |
| Gammaproteobacteria | Xanthomonadales | *Arenimonas oryziterrae* | 1.0 |
| Gammaproteobacteria | Burkholderiales | *Massilia alkalitolerans* | 1.0 |
| Alphaproteobacteria | Rhizobiales | *Bradyrhizobium sp.* | 1.0 |
| Actinomycetia | Mycobacteriales | *Actinoplanes sp000389965* | 1.0 |
| Alphaproteobacteria | Sphingomonadales | *Sphingomonas chungangi* | 1.0 |
| Alphaproteobacteria | Rhizobiales | *Mesorhizobium sp.* | 1.0 |
| Gammaproteobacteria | Burkholderiales | *Massilia kyonggiensis* | 1.0 |
| Actinomycetia | Mycobacteriales | *Rhodococcus nanhaiensis* | 1.0 |
| Alphaproteobacteria | Rhizobiales | *Neorhizobium petrolearium* | 1.0 |
| Alphaproteobacteria | Rhizobiales | *Devosia sp001427875* | 1.0 |
| Alphaproteobacteria | Sphingomonadales | *Sphingobium sp.* | 1.0 |
| Alphaproteobacteria | Azospirillales | *Lacibacterium aquatile* | 1.0 |
| Bacilli | Staphylococcales | *Staphylococcus capitis* | 1.0 |
| Alphaproteobacteria | Rhodobacterales | *Rhodobacter ruber* | 1.0 |
| Gammaproteobacteria | Enterobacterales | *Pararheinheimera texasensis* | 1.0 |
| Alphaproteobacteria | Rhodobacterales | *Gemmobacter aquatilis* | 1.0 |
| Gammaproteobacteria | Xanthomonadales | *Lysobacter panacisoli* | 1.0 |
| Alphaproteobacteria | Rhizobiales | *Aestuariivirga litoralis* | 1.0 |
| Alphaproteobacteria | Rhodobacterales | *Paracoccus haematequi* | 1.0 |
| Actinomycetia | Mycobacteriales | *Mycobacterium setense* | 1.0 |
| Alphaproteobacteria | Rhizobiales | *Mycoplana sp.* | 1.0 |
| Alphaproteobacteria | Rhizobiales | *Devosia riboflavina* | 1.0 |
| Alphaproteobacteria | Rhizobiales | *Shinella fusca* | 1.0 |
| Actinomycetia | Mycobacteriales | *Mycobacterium mucogenicum_B* | 1.0 |
| Actinomycetia | Propionibacteriales | *Nocardioides daeguensis* | 1.0 |
| Alphaproteobacteria | Sphingomonadales | *Novosphingobium resinovorum* | 1.0 |
| Bacteroidia | AKYH767 | *UBA2475 sp002319075* | 1.0 |
| Bacteroidia | AKYH767 | *UBA4416 sp002420145* | 1.0 |
| Gammaproteobacteria | Burkholderiales | *Unidentified* | 1.0 |
| Alphaproteobacteria | Sphingomonadales | *Blastomonas ursincola* | 1.0 |
| Actinomycetia | Actinomycetales | *Microbacterium marinum* | 1.0 |
| Alphaproteobacteria | Sphingomonadales | *Novosphingobium resinovorum* | 1.0 |
| Alphaproteobacteria | Azospirillales | *Aerophototrophica sp.* | 1.0 |
| Gammaproteobacteria | Burkholderiales | *Comamonas litopenaei* | 1.0 |
| Thermoleophilia | Miltoncostaeales | *Miltoncostaea oceani* | 1.0 |
| Gammaproteobacteria | Enterobacterales | *Pararheinheimera tangshanensis* | 1.0 |
| Gammaproteobacteria | Burkholderiales | *Achromobacter piechaudii* | 1.0 |
| Gammaproteobacteria | Burkholderiales | *Comamonas terrigena* | 1.0 |
| Bacteroidia | Flavobacteriales | *PHOS-HE28 sp902825795* | 1.0 |
| Gammaproteobacteria | Burkholderiales | *Ramlibacter rhizophilus* | 1.0 |
| Gammaproteobacteria | Pseudomonadales | *Alcanivorax venustensis* | 1.0 |
| Gammaproteobacteria | Pseudomonadales | *Pseudomonas sp.* | 1.0 |
| Actinomycetia | Streptomycetales | *Streptomyces canus* | 1.0 |
| Alphaproteobacteria | Caulobacterales | *Phenylobacterium haematophilum* | 1.0 |
| Gammaproteobacteria | Xanthomonadales | *Rhodanobacter spathiphylli* | 1.0 |
| Gammaproteobacteria | Enterobacterales | *Pararheinheimera tangshanensis* | 1.0 |
| Gammaproteobacteria | Enterobacterales | *Enterobacter hormaechei_712820* | 1.0 |
| Alphaproteobacteria | Caulobacterales | *Amphiplicatus metriothermophilus* | 1.0 |
| Bacteroidia | Chitinophagales | *Edaphocola coffeisoli* | 1.0 |
| Actinomycetia | Mycobacteriales | *Actinoplanes consettensis* | 1.0 |
| Alphaproteobacteria | Rhizobiales | *Shinella kummerowiae* | 1.0 |
| Gammaproteobacteria | Pseudomonadales | *Cellvibrio ostraviensis* | 1.0 |
| Gammaproteobacteria | Xanthomonadales | *Lysobacter hankyongensis* | 1.0 |
| Bacteroidia | Sphingobacteriales | *Sphingobacterium sp.* | 1.0 |
| Bacteroidia | Chitinophagales | *Unidentified* | 1.0 |
| Polyangia_463783 | Polyangiales | *Unidentified* | 1.0 |
| Alphaproteobacteria | Rhizobiales | *Roseiarcus fermentans* | 1.0 |
| Gammaproteobacteria | Burkholderiales | *Variovorax soli* | 1.0 |
| Bacteroidia | Sphingobacteriales | *Pseudosphingobacterium sp.* | 1.0 |
| Alphaproteobacteria | Rhizobiales | *Shinella kummerowiae* | 1.0 |
| Alphaproteobacteria | Rhizobiales | *Rhizobium sp.* | 1.0 |
| Alphaproteobacteria | Rhizobiales | *Rhizobium metallidurans* | 1.0 |
| Gammaproteobacteria | Enterobacterales | *Yokenella regensburgei* | 1.0 |
| Alphaproteobacteria | Rhodobacterales | *Wagnerdoeblera sp.* | 1.0 |
| Alphaproteobacteria | Sphingomonadales | *Sphingopyxis soli* | 1.0 |
| Gammaproteobacteria | Burkholderiales | *Rhodoferax sp003415675* | 1.0 |
| Gammaproteobacteria | Burkholderiales | *Hydrogenophaga soli* | 1.0 |
| Alphaproteobacteria | Caulobacterales | *Brevundimonas fluminis* | 1.0 |
| Alphaproteobacteria | Rhizobiales | *Rhizobium sp.* | 1.0 |
| Actinomycetia | Actinomycetales | *Leucobacter aerolatus* | 1.0 |
| Alphaproteobacteria | Rhizobiales | *Rhizobium tropici_500522* | 1.0 |
| Gammaproteobacteria | Xanthomonadales | *Rhodanobacter aciditrophus* | 1.0 |
| Gammaproteobacteria | Pseudomonadales | *Cellvibrio mixtus* | 1.0 |
| Alphaproteobacteria | Rhizobiales | *Bradyrhizobium sp009781045* | 1.0 |
| Alphaproteobacteria | Rhizobiales | *Rhizobium etli* | 1.0 |
| Alphaproteobacteria | Rhizobiales | *Rhizobium sp.* | 1.0 |
| Alphaproteobacteria | Rhizobiales | *Rhizobium sp.* | 1.0 |
| Gammaproteobacteria | Enterobacterales | *Escherichia coli* | 1.0 |
| Gammaproteobacteria | Pseudomonadales | *Halomonas sp.* | 1.0 |
| Alphaproteobacteria | Rhodobacterales | *Tabrizicola alkalilacus* | 1.0 |
| Alphaproteobacteria | Rhizobiales | *Rhizobium mesoamericanum* | 1.0 |
| Gammaproteobacteria | Enterobacterales | *Pararheinheimera tangshanensis* | 1.0 |
| Alphaproteobacteria | Rhizobiales | *Bradyrhizobium subterraneum* | 1.0 |
| Actinomycetia | Actinomycetales | *Agromyces terreus* | 1.0 |
| Alphaproteobacteria | Sphingomonadales | *Caenibius tardaugens* | 1.0 |
| Actinomycetia | Actinomycetales | *Leifsonia sp.* | 1.0 |
| Verrucomicrobiae | Verrucomicrobiales | *Prosthecobacter gellanilyticus* | 1.0 |
| Bacteroidia | Flavobacteriales | *Flavobacterium album_A_818019* | 1.0 |
| Alphaproteobacteria | RUG11792 | *RUG11420 sp.* | 1.0 |
| Alphaproteobacteria | Rhizobiales | *Bradyrhizobium vignae* | 1.0 |
| Gammaproteobacteria | Burkholderiales | *Comamonas sp.* | 1.0 |
| Actinomycetia | Actinomycetales | *Microbacterium rhizosphaerae* | 1.0 |
| Bacteroidia | Chitinophagales | *Edaphocola sp003852495* | 1.0 |
| Verrucomicrobiae | Pedosphaerales | *Pedosphaera parvula* | 1.0 |
| Actinomycetia | Actinomycetales | *Microbacterium aoyamense* | 1.0 |
| Actinomycetia | Streptomycetales | *Streptomyces sp.* | 1.0 |
| Actinomycetia | Mycobacteriales | *Nakamurella intestinalis* | 1.0 |
| Alphaproteobacteria | Ferrovibrionales | *Ferrovibrio terrae* | 1.0 |
| Alphaproteobacteria | Caulobacterales | *Brevundimonas kwangchunensis* | 1.0 |
| Gammaproteobacteria | Lysobacterales | *Chujaibacter soli* | 1.0 |
| Bacteroidia | Chitinophagales | *Terrimonas rhizosphaerae* | 1.0 |
| Alphaproteobacteria | Sphingomonadales | *Sphingobium chungbukense* | 1.0 |
| Thermoleophilia | Solirubrobacterales | *67-14 sp001897355* | 1.0 |
| Gammaproteobacteria | Burkholderiales | *Pseudoduganella eburnea* | 1.0 |
| Bacteroidia | Chitinophagales | *Flavihumibacter cheonanensis* | 1.0 |
| Alphaproteobacteria | Ferrovibrionales | *Ferrovibrio sp002796975* | 1.0 |
| Alphaproteobacteria | Rhizobiales | *Rhizobium sp.* | 1.0 |
| Alphaproteobacteria | Rhizobiales | *Ochrobactrum pseudintermedium* | 1.0 |
| Alphaproteobacteria | Sphingomonadales | *Sphingomonas jinjuensis* | 1.0 |
| Bacteroidia | Sphingobacteriales | *Pedobacter nyackensis* | 1.0 |
| Actinomycetia | Mycobacteriales | *Actinoplanes lichenis* | 1.0 |
| Alphaproteobacteria | Rhizobiales | *Bradyrhizobium sp.* | 1.0 |
| Thermoleophilia | Solirubrobacterales | *Solirubrobacter pauli* | 1.0 |
| Alphaproteobacteria | Rhizobiales | *Devosia honganensis* | 1.0 |
| Alphaproteobacteria | Sphingomonadales | *Sphingobium fuliginis* | 1.0 |
| Alphaproteobacteria | Rhizobiales | *Bradyrhizobium guangdongense* | 1.0 |
| Bacteroidia | Cytophagales | *Dyadobacter ginsengisoli* | 1.0 |
| Alphaproteobacteria | Dongiales | *Dongia mobilis* | 1.0 |
| Gammaproteobacteria | Pseudomonadales | *Pseudomonas borbori* | 1.0 |
| Alphaproteobacteria | Rhizobiales | *Neorhizobium pakistanense* | 1.0 |
| Alphaproteobacteria | Caedimonadales | *Paracaedimonas acanthamoebae* | 1.0 |
| Actinomycetia | Mycobacteriales | *Lawsonella sp.* | 1.0 |
| Alphaproteobacteria | Rhizobiales | *Bosea sp.* | 1.0 |
| Alphaproteobacteria | N/A | *Unidentified* | 1.0 |
| Actinomycetia | Mycobacteriales | *Rhodococcus fascians_E_375049* | 1.0 |
| Bacteroidia | NS11-12g_892060 | *Unidentified* | 1.0 |
| Bacteroidia | Flavobacteriales | *Chryseobacterium taichungense* | 1.0 |
| Alphaproteobacteria | Rhizobiales | *Pseudolabrys sp001426945* | 1.0 |
| Gammaproteobacteria | Burkholderiales | *Achromobacter mucicolens* | 1.0 |
| Gammaproteobacteria | Burkholderiales | *Paucibacter sp001477625* | 1.0 |
| Bacteroidia | Cytophagales | *Dyadobacter endophyticus* | 1.0 |
| Alphaproteobacteria | Rhizobiales | *Hyphomicrobium denitrificans_496618* | 1.0 |
| Verrucomicrobiae | Verrucomicrobiales | *Luteolibacter sp.* | 1.0 |
| Alphaproteobacteria | Rhizobiales | *Shinella kummerowiae* | 1.0 |
| Alphaproteobacteria | Sphingomonadales | *Sphingobium vulgare* | 1.0 |
| Actinomycetia | Mycobacteriales | *Pseudonocardia sp.* | 1.0 |
| Alphaproteobacteria | Rhizobiales | *Methyloterrigena soli* | 1.0 |
| Alphaproteobacteria | Sphingomonadales | *Sphingobium chlorophenolicum* | 1.0 |
| Gammaproteobacteria | Xanthomonadales | *Xanthomonas albilineans* | 1.0 |
| Bacteroidia | Sphingobacteriales | *Sphingobacterium sp.* | 1.0 |
| Bacteroidia | Chitinophagales | *Niabella hibiscisoli* | 1.0 |
| Gammaproteobacteria | Burkholderiales | *Brachymonas chironomi* | 1.0 |
| Gammaproteobacteria | Xanthomonadales | *Stenotrophomonas sp.* | 1.0 |
| Gammaproteobacteria | Burkholderiales | *Pseudorhodoferax caeni* | 1.0 |
| Gammaproteobacteria | Enterobacterales | *Pararheinheimera texasensis* | 1.0 |
| Bacteroidia | Cytophagales | *Amoebophilus asiaticus* | 1.0 |
| Paceibacteria | Paceibacterales | *GWA2-37-10* | 1.0 |
| Gammaproteobacteria | Burkholderiales | *Methyloversatilis thermotolerans* | 1.0 |
| Gammaproteobacteria | Burkholderiales | *Pseudorhodoferax aquiterrae* | 1.0 |
| Bacteroidia | Flavobacteriales | *Flavobacterium limi* | 1.0 |
| Gammaproteobacteria | Burkholderiales | *Acidovorax oryzae* | 1.0 |
| Alphaproteobacteria | Rhizobiales | *Devosia sediminis* | 1.0 |
| Limnochordia | DTU010 | *DTU012* | 1.0 |
| Bacteroidia | Flavobacteriales | *Flavobacterium amnigenum* | 1.0 |
| Fibrobacteria | Fibrobacterales | *Unidentified* | 1.0 |
| Gammaproteobacteria | Burkholderiales | *Comamonas terrigena* | 1.0 |
| Bacteroidia | Chitinophagales | *Terrimonas rubra* | 1.0 |
| Alphaproteobacteria | Caulobacterales | *Caulobacter sp.* | 1.0 |
| Gammaproteobacteria | Enterobacterales | *Lelliottia jeotgali* | 1.0 |
| Gammaproteobacteria | Xanthomonadales | *Lysobacter agri* | 1.0 |
| Bacteroidia | Cytophagales | *Ohtaekwangia sp.* | 1.0 |
| Verrucomicrobiae | Chthoniobacterales | *Terrimicrobium sacchariphilum* | 1.0 |
| Alphaproteobacteria | Rhizobiales | *Bosea psychrotolerans* | 1.0 |
| Bacteroidia | Cytophagales | *Arcicella rosea* | 1.0 |
| Alphaproteobacteria | Rhizobiales | *Rhizobium pseudoryzae* | 1.0 |
| Alphaproteobacteria | Rhizobiales | *Rhizobium rhizoryzae* | 1.0 |
| Actinomycetia | Mycobacteriales | *Actinokineospora mzabensis* | 1.0 |
| Bacteroidia | Chitinophagales | *Agriterribacter humi* | 1.0 |
| Actinomycetia | Streptomycetales | *Streptomyces prunicolor* | 1.0 |
| Gammaproteobacteria | DSM-16500 | *Aquicella sp.* | 1.0 |
| Alphaproteobacteria | Sphingomonadales | *Sphingomonas aquatilis* | 1.0 |
| Bacteroidia | Flavobacteriales | *Chryseobacterium camelliae* | 1.0 |
| Gammaproteobacteria | Burkholderiales | *Comamonas tsuruhatensis* | 1.0 |
| Actinomycetia | Mycobacteriales | *Pseudonocardia hydrocarbonoxydans* | 1.0 |
| Bacteroidia | Cytophagales | *Dyadobacter sp.* | 1.0 |
| Alphaproteobacteria | Sphingomonadales | *Rhizorhabdus histidinilytica* | 1.0 |
| Bacteroidia | Chitinophagales | *Edaphocola yonginensis* | 1.0 |
| Gammaproteobacteria | Burkholderiales | *Acidovorax delafieldii_B_589608* | 1.0 |
| Alphaproteobacteria | Rhizobiales | *28-YEA-48 sp900104955* | 1.0 |
| Bacteroidia | Flavobacteriales | *Flavobacterium sp004801375* | 1.0 |
| Bacteroidia | Chitinophagales | *Sediminibacterium goheungense* | 1.0 |
| Gammaproteobacteria | Burkholderiales | *Methylophilus flavus* | 1.0 |
| Gammaproteobacteria | Xanthomonadales | *Rhodanobacter denitrificans* | 1.0 |
| Alphaproteobacteria | Caulobacterales | *Caulobacter vibrioides_C_487720* | 1.0 |
| Babeliae | Babeliales | *Chromulinavorax destructans* | 1.0 |
| Gammaproteobacteria | Burkholderiales | *Pseudorhodoferax soli* | 1.0 |
| Bacteroidia | Flavobacteriales | *Chryseobacterium lathyri* | 1.0 |
| Alphaproteobacteria | Rhizobiales | *Bosea caraganae* | 1.0 |
| Gammaproteobacteria | Pseudomonadales | *Atopomonas hussainii* | 1.0 |
| Gammaproteobacteria | Pseudomonadales | *Pseudomonas sp.* | 1.0 |
| Paceibacteria | Paceibacterales | *GWA2-37-10* | 1.0 |
| Bacteroidia | Sphingobacteriales | *Sphingobacterium mizutaii* | 1.0 |
| Gammaproteobacteria | Pseudomonadales | *Pseudomonas chengduensis* | 1.0 |
| Bacteroidia | Cytophagales | *ELB16-189* | 1.0 |
| Bacteroidia | Flavobacteriales | *Chryseobacterium sp.* | 1.0 |
| Gammaproteobacteria | Pseudomonadales | *Cellvibrio sp.* | 1.0 |
| Alphaproteobacteria | Rhizobiales | *Rhizobium sp.* | 1.0 |
| Alphaproteobacteria | Sphingomonadales | *Sphingobium scionense* | 1.0 |
| Gammaproteobacteria | Steroidobacterales | *Steroidobacter sp.* | 1.0 |

**Supplementary Table 3.** Homogeneous groups in fungal and bacterial community abundance of the six genotypes of tomato (ADX2, ISR-10, MO-10, TH-30, AIL, MM). Results obtained with LSD-Fisher method for p<0.05.

|  | Ascomycota | Basidiomycota | Chytridiomycota | Mortierellomycota | Mucoromycota | NA |
| --- | --- | --- | --- | --- | --- | --- |
| ADX2 | a | a | a | a | a | ab |
| ISR-10 | abc | b | a | b | ab | b |
| MO-10 | abc | b | a | ab | b | a |
| TH-30 | ab | ab | a | ab | a | ab |
| AIL | c | b | a | b | ab | b |
| MM | bc | ab | a | ab | ab | ab |

|  | Actinomycetia | Bacteroidia | Chloroflexia | Gammaproteobacteria | Alphaproteobacteria |
| --- | --- | --- | --- | --- | --- |
| ADX2 | ab | b | a | ab | a |
| ISR-10 | ab | b | a | ab | a |
| MO-10 | ab | ab | a | ab | a |
| TH-30 | a | a | a | a | a |
| AIL | b | b | a | b | a |
| MM | b | b | a | b | a |

**Supplementary Table 4.** Homogeneous groups in fungal and bacterial community abundance of the four genotypes of tomato (ADX2, TH-30, AIL, MM) used for fungicide treatment and mock treatment (control). Results obtained with LSD-Fisher method for p<0.05.

|  | Ascomycota | Basidiomycota | Chytridiomycota | Mortierellomycota | Mucoromycota |
| --- | --- | --- | --- | --- | --- |
| ADX2 (control) | b | b | a | a | a |
| AIL (control) | c | b | a | b | a |
| MM (control) | bc | b | a | ab | a |
| TH-30 (control) | bc | b | a | b | a |
| ADX2 (treated) | bc | b | a | b | a |
| AIL (treated) | bc | b | a | ab | a |
| MM (treated) | bc | b | a | ab | a |
| TH-30 (treated) | a | a | a | ab | a |

|  | Actinomycetia | Bacteroidia | Chloroflexia | Gammaproteobacteria | Alphaproteobacteria |
| --- | --- | --- | --- | --- | --- |
| ADX2 (control) | b | abc | b | b | ab |
| AIL (control) | b | c | b | c | ab |
| MM (control) | b | c | b | bc | b |
| TH-30 (control) | b | ab | b | bc | ab |
| ADX2 (treated) | b | bc | b | bc | ab |
| AIL (treated) | b | abc | b | bc | ab |
| MM (treated) | b | c | b | bc | ab |
| TH-30 (treated) | a | a | a | a | a |

**Supplementary Table 5.** Abundance increases of bacterial taxa in each tomato variety (ADX2, TH-30, AIL, MM) under fungicide treatment. The numbers indicate the taxa that showed a higher relative abundance compared to the control after fungicide treatment.

| **Bacterial Order** | **ADX2** | **TH-30** | **AILSA** | **MM** |
| --- | --- | --- | --- | --- |
| Chitinophagales | 10 | 37 | 22 | 9 |
| Azospirillales_507929 | 0 | 5 | 3 | 4 |
| Enterobacterales_A_737866 | 2 | 17 | 20 | 10 |
| Rhizobiales_A_501058 | 7 | 11 | 9 | 4 |
| Sphingomonadales | 5 | 17 | 22 | 8 |
| Steroidobacterales | 1 | 8 | 2 | 3 |

| **Fungal Order** | **ADX2** | **TH-30** | **AILSA** | **MM** |
| --- | --- | --- | --- | --- |
| Atheliales | 2 | 1 | 1 | 0 |
| Agaricales | 0 | 0 | 1 | 0 |
| Atractiellales | 0 | 1 | 1 | 0 |
| Cantharellales | 2 | 0 | 1 | 0 |
| Chaetothyriales | 1 | 1 | 5 | 2 |
| Capnodiales | 4 | 0 | 3 | 0 |
| Classiculales | 1 | 1 | 1 | 1 |
| Cystofilobasidiales | 2 | 0 | 2 | 0 |
| Eurotiales | 4 | 3 | 5 | 4 |
| Filobasidiales | 1 | 0 | 1 | 2 |
| Helotiales | 6 | 3 | 9 | 18 |
| Glomerellales | 1 | 3 | 4 | 1 |
| Hypocreales | 9 | 10 | 22 | 0 |
| Holtermanniales | 0 | 1 | 1 | 0 |
| Malasseziales | 2 | 1 | 2 | 0 |
| Leucosporidiales | 1 | 0 | 1 | 0 |
| Microascales | 0 | 2 | 3 | 0 |
| Myrmecridiales | 0 | 0 | 1 | 0 |
| Mortierellales | 4 | 3 | 5 | 0 |
| Platygloeales | 0 | 0 | 1 | 0 |
| Pezizales | 5 | 3 | 4 | 1 |
| Pleosporales | 4 | 2 | 7 | 13 |
| Polyporales | 1 | 0 | 2 | 0 |
| Pyxidiophorales | 0 | 1 | 1 | 0 |
| Rhizophydiales | 2 | 0 | 0 | 0 |
| Sordariales | 5 | 4 | 9 | 9 |
| Thelephorales | 1 | 2 | 2 | 0 |
| Thelebolales | 2 | 0 | 1 | 0 |
| Trechisporales | 0 | 3 | 3 | 1 |
| Trichosporonales | 0 | 0 | 1 | 0 |
| Umbelopsidales | 0 | 0 | 1 | 0 |
| unidentified | 0 | 1 | 1 | 0 |

**Supplementary Table 6.** Abundance increases of fungal taxa in each tomato variety (ADX2, TH-30, AIL, MM) under fungicide treatment. The numbers indicate the taxa that showed a higher relative abundance compared to the control after fungicide treatment.


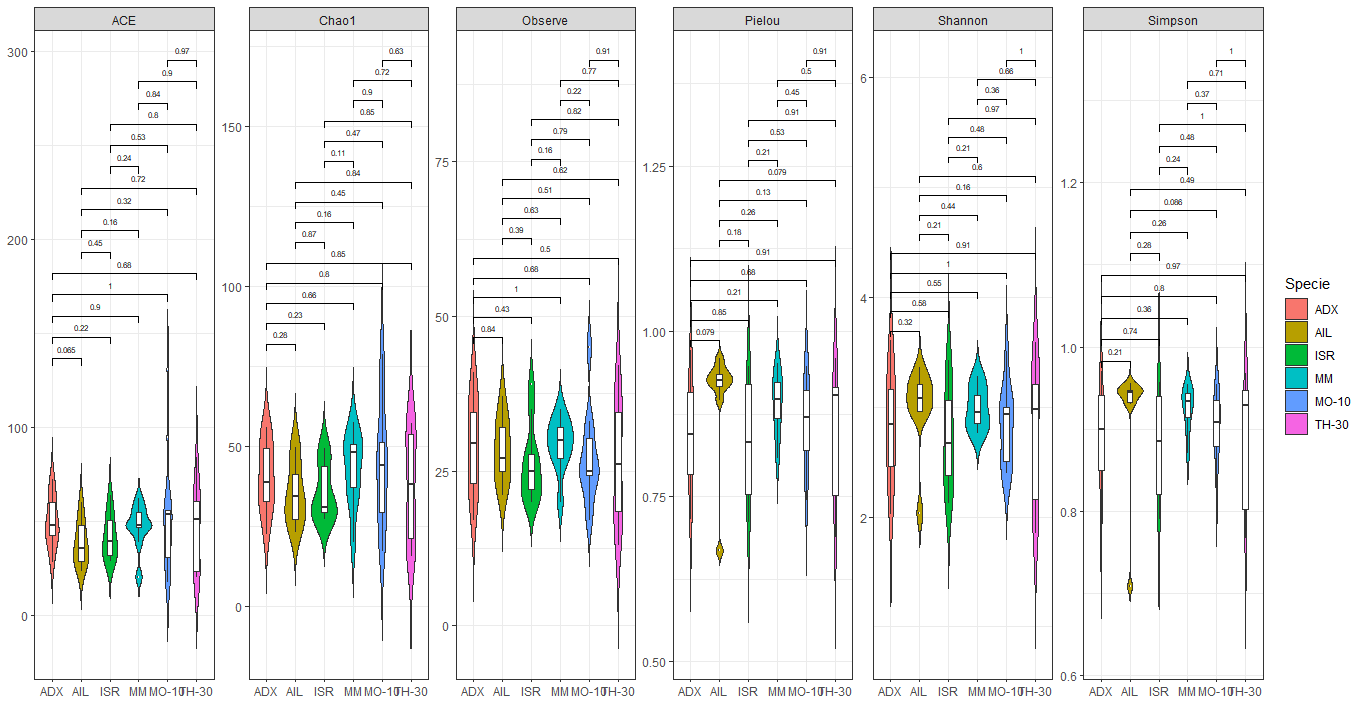


**Supplementary Figure 1.** Estimated richness of four traditional varieties (ADX2, TR-30, ISR-10, MO-10) and two commercial cultivars (AIL, MM) of *S. lycopersicum* by several diversity tests for fungal communities. The calculated diversity index corresponds to Observe, Chao1, ACE, Shannon, Simpson and J test with a P-value based on the Wilcoxon test (n=10).


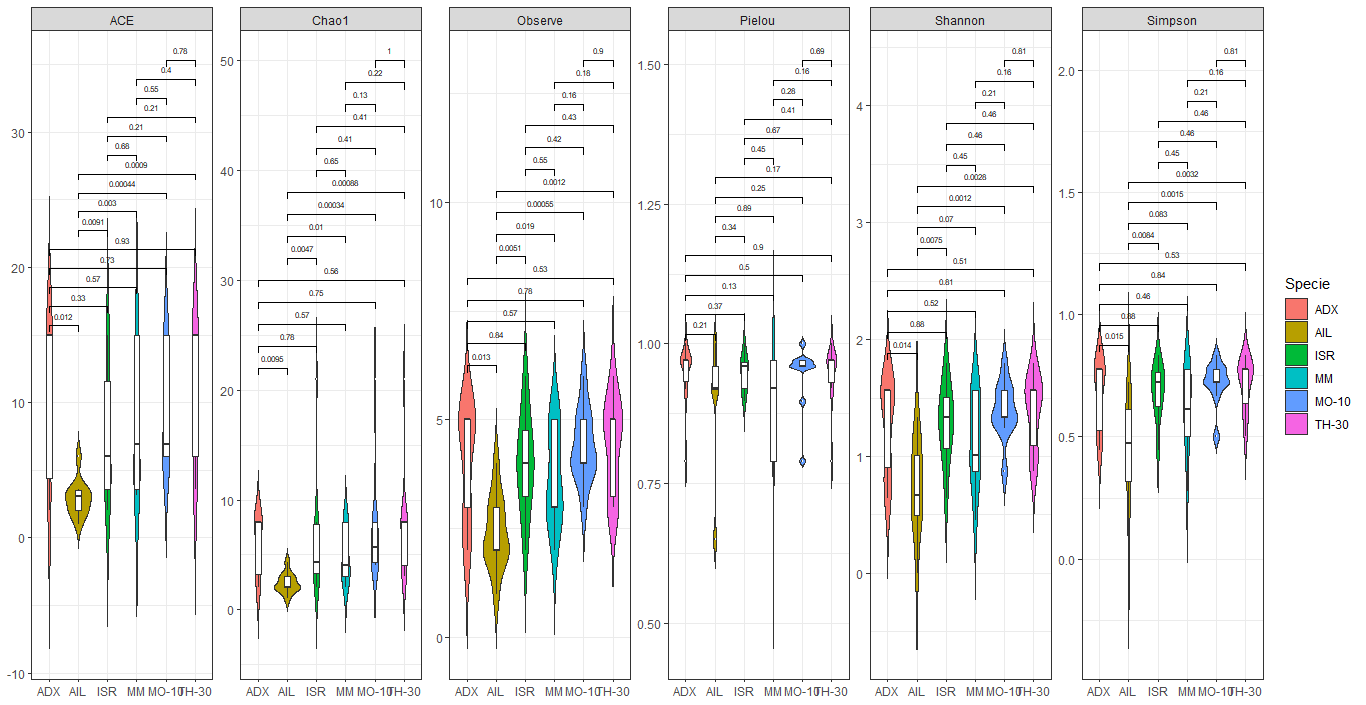


**Supplementary Figure 2.** Estimated richness of four traditional varieties (ADX2, TR-30, ISR-10, MO-10) and two commercial cultivars (AIL, MM) of *S. lycopersicum* by several diversity tests for bacterial communities. The calculated diversity index corresponds to Observe, Chao1, ACE, Shannon, Simpson and J test with a P-value based on the Wilcoxon test (n=10).
